# Photocharging of Carbon Nitride Thin Films for Controllable Manipulation of Droplet Force Gradient Sensors

**DOI:** 10.1021/jacs.3c09084

**Published:** 2023-11-07

**Authors:** Bradley D. Frank, Markus Antonietti, Paolo Giusto, Lukas Zeininger

**Affiliations:** Department of Colloid Chemistry, Max Planck Institute of Colloids and Interfaces, Am Mühlenberg 1, 14476 Potsdam, Germany

## Abstract

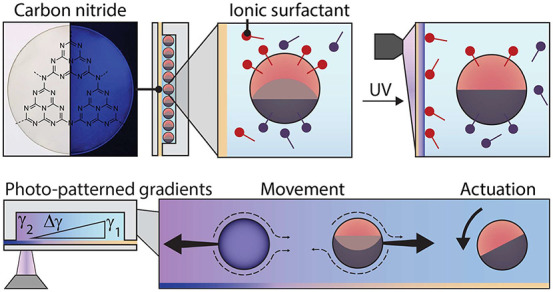

Intentional generation, amplification, and discharging of chemical gradients is central to many nano- and micromanipulative technologies. We describe a straightforward strategy to direct chemical gradients inside a solution via local photoelectric surface charging of organic semiconducting thin films. We observed that the irradiation of carbon nitride thin films with ultraviolet light generates local and sustained surface charges in illuminated regions, inducing chemical gradients in adjacent solutions via charge-selective immobilization of surfactants onto the substrate. We studied these gradients using droplet force gradient sensors, complex emulsions with simultaneous and independent responsive modalities to transduce information on transient gradients in temperature, chemistry, and concentration via tilting, morphological reconfiguration, and chemotaxis. Fine control over the interaction between local, photoelectrically patterned, semiconducting carbon nitride thin films and their environment yields a new method to design chemomechanically responsive materials, potentially applicable to micromanipulative technologies including microfluidics, lab-on-a-chip devices, soft robotics, biochemical assays, and the sorting of colloids and cells.

Harnessing energy gradients to prompt mechanical responses is an essential attribute that enables microorganisms to live, persist, and perform essential biological functions. In nature, various energy gradients are instrumentalized to drive nano- to microscale processes, including molecular transport mediated by motor proteins as well as diffusiophoretic and electrophoretic reactions of whole cellular ensembles.^1−3^ Synthetic efforts to emulate these behaviors target the design of active dissipative material systems that autonomously regulate their motile behaviors in response to their immediate chemical environment.^4−8^

Various nature-inspired active colloidal systems convert chemical concentration gradients into mechanical energy as a basis to control motile, interactive, and collective behaviors.^9−11^ Despite broad applications,^12−14^ available techniques are limited to methods that require elaborate and sophisticated substrate design for surface-encoded transportation schemes,^15−19^ involve intrusive anisotropic surface modification of colloids,^20−22^ or require an intensive energy input.^23−26^ Simple generalizable methods to generate chemical gradients are a compelling scientific need, where a non-destructive, reversible, and selective evocation of spatiotemporally controlled chemical gradients inside a solution has not yet been reported.

With the long-term goal of establishing a broadly applicable and energy-efficient manipulation technique for directing nano- and micro-objects inside a solution, we report a new strategy for in situ and reversible evocation of chemical gradients. We anticipated that reversible adsorption of molecular solutes via controllable photopatterning of semiconductor substrates could evoke chemical gradients inside a solution.

Carbon nitride is a metal-free semiconducting polymer, and its structure primarily consists of 2D graphitic-like planes of cross-linked heptazine units (Figure 1a), which causes anisotropy of electronic transport, enabling long-lived surface charges. High extinction coefficients in the UV range^27^ enable splitting electrons and holes between thin layers,^28,29^ resulting in a collective net charge by graphitic planes.^30,31^ Upon UV illumination of a carbon nitride-coated glass slide covered with an aqueous sodium dodecyl sulfate (SDS) surfactant solution, looking through an air bubble, we observed rapid de-wetting and spreading of the bubble occurring exclusively within the illuminated areas of the sample (Figure 1c). In the same experiment, without any surfactant present, no response is observed (Movie S1). As polymeric carbon nitride thin films are hydrophilic,^32^ we attributed this observation to photogenerated surface charges, which, in turn, caused an adsorption of surfactants to the charged surface, consequently inducing a triggered hydrophobicity to the carbon nitride substrate (Figure 1c). The de-wetting occurred rapidly, on the order of seconds, and exclusively with ionic surfactant and in the illuminated areas of the substrates. When the UV light was removed, the reverse process occurred on the order of minutes (Figure 1c, Movie S1).

**Figure 1:**
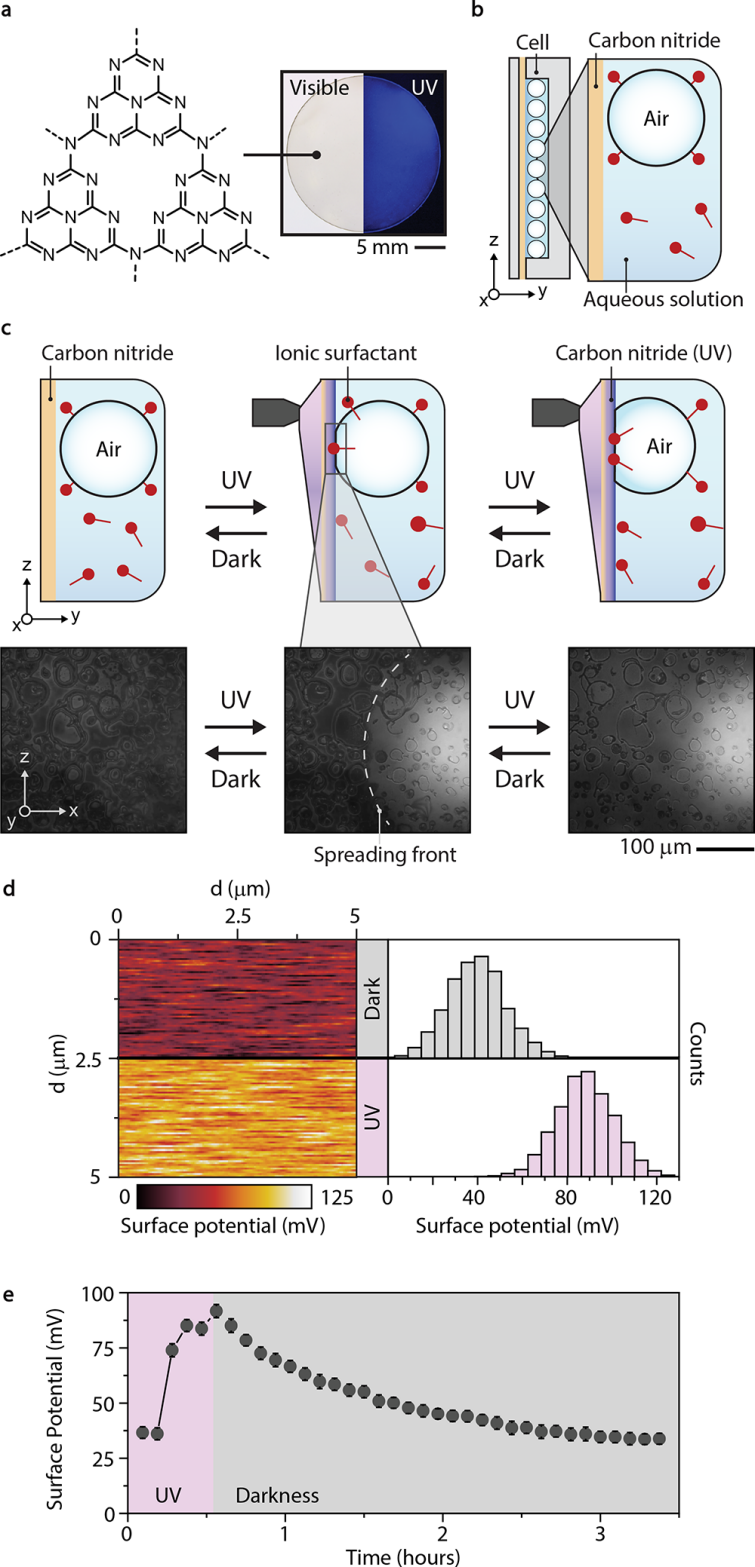
(a) Optical micrograph of carbon nitride thin film on a glass surface under visible and UV light. (b) Scheme of sample holder. (c) Scheme for carbon nitride thin film and chamber, where air bubbles sit in ionic surfactant, reversibly wetting a carbon nitride thin film, with through-bubble optical micrographs showing light-induced de-wetting of a carbon nitride thin film with surfactant and bubble spreading. (d) KPFM measurements of carbon nitride surface potential before and after the application of UV light. (e) Time-resolved KPFM measurements of carbon nitride surface charging averaged over a 5 × 5 μm^2^ area.

To quantify UV-light-induced charging of carbon nitride thin films, we performed time-resolved Kelvin probe force microscopy (KPFM) measurements. In ambient conditions, the carbon nitride thin film showed a surface potential difference when illuminated with UV light of +60 mV, above the surface potential reported for aqueous SDS adsorption (Figure 1d, Figure S1).^33,34^

Time-resolved measurements revealed that saturation charging of the film occurred on the order of minutes, while discharging proceeded over 3 h in darkness (Figure 1e). Spatially controlled absorption of UV light by the carbon nitride and associated immobilization of surfactants to the substrate induce variations in surfactant concentration, surfactant composition, and temperature.

To monitor these gradients inside the aqueous medium, we introduce droplet force gradient sensors that display a series of separate responsive modes as a tool to visualize transient gradients in situ. Droplet force gradient sensors are surfactant-stabilized complex emulsion droplets that morphologically reconfigure, triggered by characteristic^35^ and programmable^36^ changes in surfactant concentration, with up to femtomolar chemical sensitivity,^37,38^ move chemotactically in response to interfacial tension differentials,^39^ and tilt out of gravitational alignment, caused by intra-droplet thermocapillary fluid convections.^40^

We employed complex emulsion droplets composed of a phase-separated mixture of decane and methoxyperfluorobutane stabilized by hydrocarbon and fluorocarbon surfactants, namely SDS and Zonyl FS-300 (Zonyl), respectively (Figure 2a). Changes in internal droplet morphology are characteristic to variations in the balance of interfacial tensions between either phase and the continuous aqueous medium, and variations in the surfactant concentration are quantitatively described by the contact angle, θ (Figure 2a,b).^38^ Irradiating the substrates with UV light (LED, 365 nm) resulted in a net change of Δθ = 14° to the complex-droplet morphology (Figure 2c,d), which reversed when UV light was removed and the sample was left in darkness, indicating that the concentration of surfactant in the solution had returned to its initial state. The reconfiguration occurred over 3 h, in agreement with the reversible charging observed via KFPM (Figure 2d).

**Figure 2:**
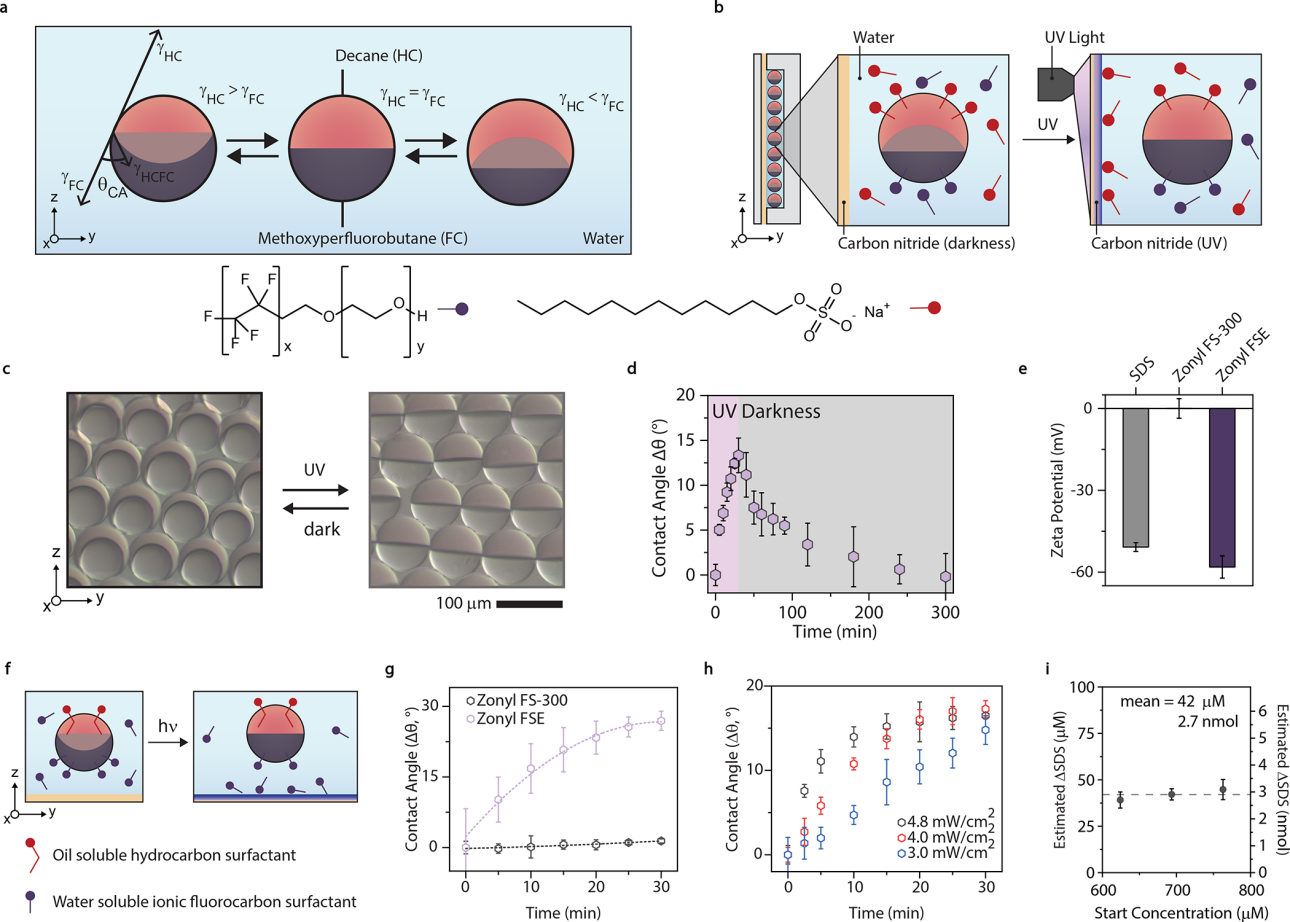
(a) Complex emulsions are shape-tunable based on interfacial tension equilibrium. (b) Schematic of droplet reconfiguration via adsorbing surfactant (sodium dodecyl sulfate, SDS) from solution, changing the balance of interfacial tensions. (c) Side-view optical micrographs of droplets composed of decane and methoxyperfluorobutane, stabilized by SDS and Zonyl, actuating in response to photocharging of a carbon nitride thin film. (d) Time-resolved morphological reconfiguration of complex droplets. (e) Micellar zeta-potential of surfactants used in this study. (f) Schematic for the change of morphology for droplets with one external and one internal surfactant. (g) Change in contact angle over time for droplets stabilized by equal concentrations of Zonyl FS-300 vs Zonyl FSE. (h) Light-intensity-dependent changes in droplet contact angle over time. (i) Change in solution concentration of SDS calculated from droplet morphology change at varying starting concentrations.

To separate the response of surfactants in solution depending on their electrophoretic mobility, we stabilized complex emulsions using an aqueous surfactant and a droplet-contained oil-soluble non-ionic surfactant (Span 20). With only one surfactant in the aqueous phase available to the substrate, we can distinguish the response to surface charging of either the non-ionic Zonyl FS-300 or ionic Zonyl FSE (ammonium bis[2-(perfluoroalkyl)ethyl] phosphate) (Figure 2e,f). When we irradiated the substrate, only droplets in the ionic surfactant solution were observed to reconfigure (Figure 2g), indicating charge selectivity. By varying the intensity of light delivered to the substrate, we could tune the rate of target solute immobilization to the substrates, which ultimately affects the droplet morphology (Figure 2h). By monitoring droplet morphology over time at different concentrations, we verified an adsorption limit to the substrate after approximately 30 min, immobilizing the same number of surfactants regardless of starting concentration (Figure 2i).

Next, we monitored the droplets’ response to transient gradients in surfactant concentrations upon illumination of carbon nitride thin films using a focused light spot (Figure 3a, Figure S2). By confining UV-illuminated areas, the adsorption and desorption of surfactants to the carbon nitride surface introduced local surfactant concentration gradients, evoking interfacial tension differentials across the droplet interfaces (Figure 3a) and generating movement proportional to the magnitude and direction of the gradient and the surface upon which it acts.^41^ When placing monodisperse single-phase droplets stabilized by anionic surfactants (dioctyl sulfosuccinate sodium salt, AOT) on the carbon nitride films, we observed a mass migration of droplets, driven by interfacial Marangoni-type fluid flows, toward the light source over the entire sample (Figure 3a,b). The gradient radius was up to 7 mm, and droplet velocities proportional to distance indicated larger gradient differentials farther away from the spot (Figure S5), indicating higher surfactant concentration around the light spot, versus direct charge interaction between surface charge and droplet.^42^

**Figure 3:**
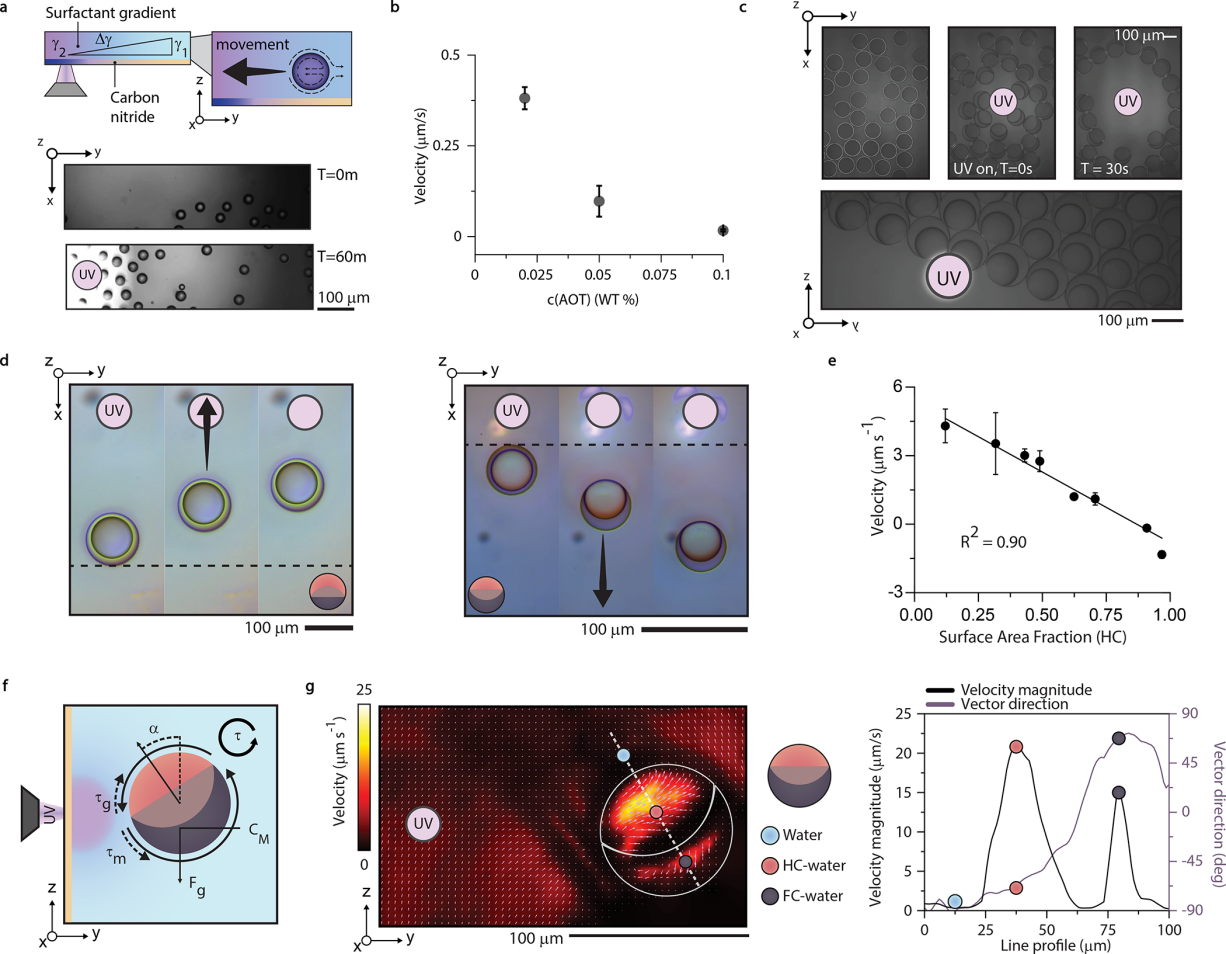
(a) Scheme to generate interfacial tension gradients on a carbon nitride thin film, inducing Marangoni flows, with inset micrographs of droplets before and after the anisotropic application of light to a carbon nitride thin film. (b) Mean velocity versus concentration of ionic hydrocarbon surfactant for droplets composed of decane:bromohexane (1:3) on a carbon nitride thin film in uniform lighting conditions. Error bars represent *n* > 10 droplets at a uniform distance over 15 min. (c) Time-series micrographs of complex droplets on carbon nitride, moving in response to a photoinduced gradient, with inset side-view micrograph of UV-induced tilting. (d) Time-series micrographs of a hydrocarbon-dominant complex emulsion droplet moving toward the light spot and a fluorocarbon-dominant complex emulsion droplet moving away from the light spot. (e) Droplet velocity versus the surface area fraction of one droplet phase for complex emulsion droplets moving on the carbon nitride thin film. Error bars represent *n* = 5 separate experiments of separated droplets at uniform distances over 15 s. (f) Scheme for Marangoni convection-induced tilting out of gravitational alignment. (g) Tilting and particle image velocimetry for a complex droplet in SDS:Zonyl exhibiting anisotropic flows, with line profile of the velocity magnitude and vector direction, noting the bulk phase, hydrocarbon–water, and fluorocarbon–water locations.

We next exposed complex droplets with a contact angle of 30° to the same experimental conditions and observed a chemotactic motion in the opposite direction from single-phase droplets and tilting out of alignment with gravity (Figure 3c). Concentration gradients induce two competitive anisotropic Marangoni flows across the external interfaces of complex droplets, where the surface area of each interface dictates the speed and direction of the droplet. Consequently, these droplets, with a fluorocarbon-dominant morphology, chemotactically moved away from the light source (Figure 3d). Conversely, complex emulsion droplets with hydrocarbon-dominant morphologies moved toward the light source. Tuning droplet morphology solely by varying the concentration of fluorocarbon surfactant (constant SDS concentration), the speed and the direction of the droplet could be controlled (Figure 3e). The existence of opposing interfacial tension gradients across the droplet was verified with side-view particle image velocimetry, where flows adjacent to either droplet interface point in opposite directions (Figure 3g, Movie S5).^43^ These external flows act competitively on the droplets, enabling morphology-reversible chemotaxis. Accompanying motion, anisotropic fluid flows cause a torque (τ) with respect to the droplet center of gravity (*C*_M_), resulting in repeatable distance-dependent actuation of the droplets (Figure 3f, Figure S6). Anisotropic tilting behaviors observed for various droplet morphologies, as well as tilting behaviors in different surfactant combinations, indicated that inter-droplet flows act constructively or destructively with heat-induced thermocapillary fluid convections from light absorption of the substrate (Figures S6 and S7).

The ability to alter concentrations reversibly with directed charging of carbon nitride thin films is immediately applicable for the micromanipulation of droplets. Without the need for target-designed stimuli-responsive surfactants, this system can push and pull droplets (Figure 4 a,b). As a tool to study the complicated dynamics of moving body systems, carbon nitride films present themselves as a non-invasive, surfactant-tunable, and reversible system. Complementing existing methods, this non-destructive system adds a further ability to study the flow dynamics of out-of-equilibrium systems. In addition, the ability to use film charging for droplet actuation is applicable to micro-optical manipulation, toward soft-matter-based displays with morphology-dependent output. By using droplets which are morphology-tuned to emit structural color dependent on morphology,^44^ charging of the carbon nitride can be used to generate UV-light-dependent structural color (Figure 4c).

**Figure 4:**
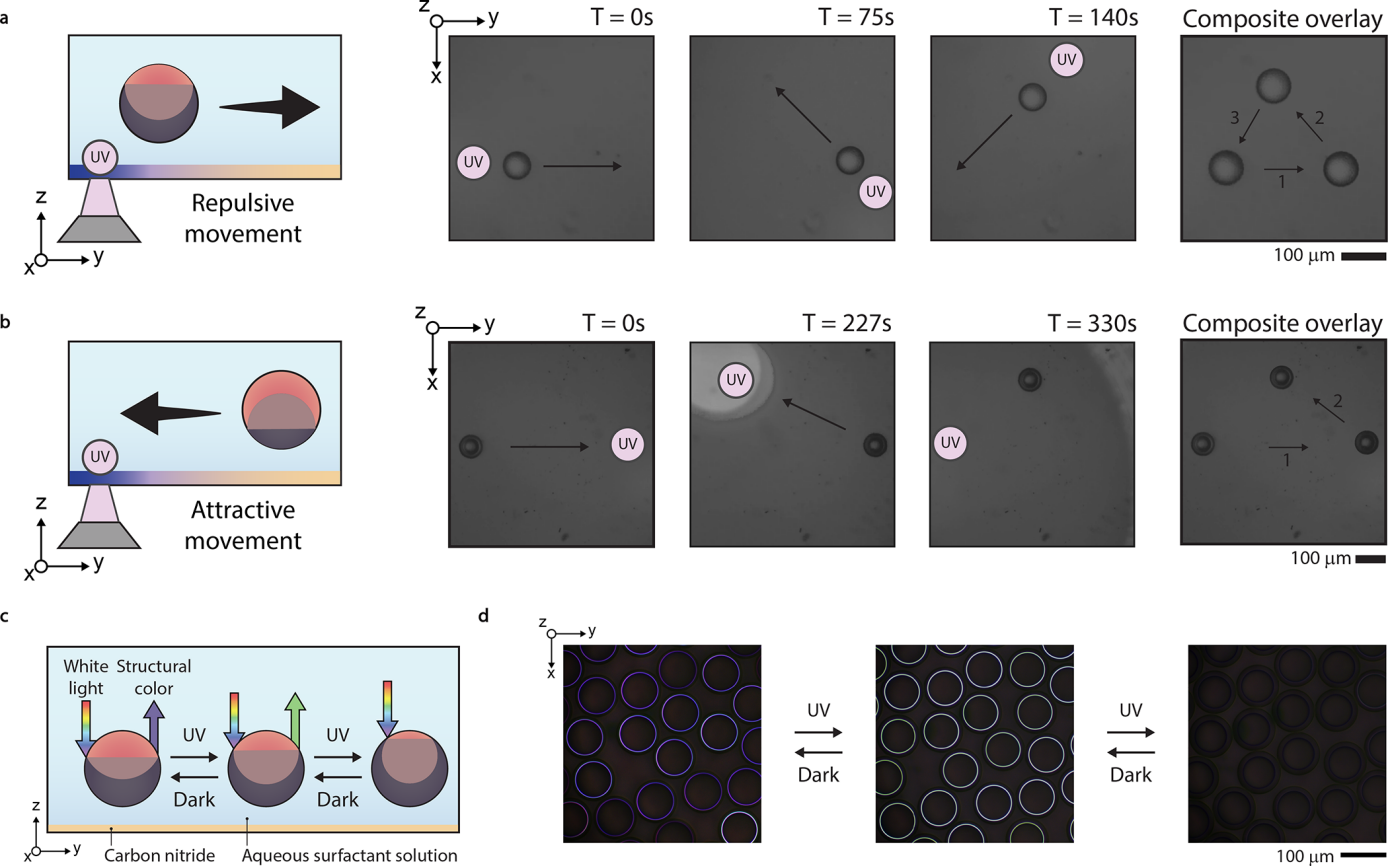
(a) Object manipulation demonstrated by pushing a fluorocarbon-dominant droplet, where the light spot was moved between frames at the indicated spot. (b) Object manipulation demonstrated by pulling a hydrocarbon-dominant droplet, where the light spot was moved to the indicated spot. (c) Optical micrographs of tunable color generation via the interaction between charged carbon nitride thin films and structurally colored complex emulsion droplets.

In conclusion, we present photoelectric charging of carbon nitride thin films directed by ultraviolet illumination, where the charge follows the illumination time and intensity. The method to translate the charge distribution into induced actuation of micrometer-scale emulsion droplets involves the reversible and charge-selective adsorption of charged solutes onto this polymeric, semiconducting substrate. Evoked gradients in temperature, surfactant concentration, and composition were studied using droplet force gradient sensors, where complex emulsions independently transduce information on the three involved transient gradients simultaneously. The herein revealed mechanistic insights into the light-triggered generation of radial millimeter-scale chemical gradients using organic semiconductors are expected to find abundant applications well beyond the demonstrated micromanipulation of artificial colloids, including in vitro manipulation of biological micro-objects such as cells, for aerosol remediation, or for an enhancement of charge carrier mobilities in electrochemical applications.

## Supporting Information Available

Materials and methods, supporting figures, and descriptions of supporting movies (PDF)
Supporting movies S1–S6 (ZIP)
