# Supplementary material for: Photocharging of Carbon Nitride Thin Films for Controllable Manipulation of Droplet Force Gradient Sensors
Source: J Am Chem Soc. 2023 Nov 7;145(45):24476–81. doi: 10.1021/jacs.3c09084 (PMC10655103; doi:10.1021/jacs.3c09084)
Supplement: Supplementary file 1 — ja3c09084_si_001.pdf [file ja3c09084_si_001.pdf]

Supporting Information

Photocharging of carbon nitride thin films for controllable manipulation of droplet force gradient sensors

Bradley D. Frank, Markus Antonietti, Paolo Giusto,* and Lukas Zeininger*

Department of Colloid Chemistry, Max Planck Institute of Colloids and Interfaces

Am Mühlenberg 1, 14476 Potsdam, Germany

Materials and Methods 2

1. Chemicals 2
2. Characterization 2
3. Deposition of carbon nitride films 2
4. Generation of complex emulsion droplets 2
5. Full-film excitation of carbon nitride substrate 3
6. Anisotropic excitation of carbon nitride substrate 3
7. Kelvin probe force microscopy 3
8. Droplet movement and flow characterization 4

Supporting figures 6

9. Supporting figure 1. 6
10. Supporting figure 2. 7
11. Supporting figure 3 8
12. Supporting figure 4. 8
13. Supporting figure 5. 9
14. Supporting figure 6. 10
15. Supporting figure 7. 11
16. Supporting figure 8. 12
17. Supporting figure 9. 13

References 14

Supporting Movies 15

Materials and Methods

Chemicals

Decane (Sigma Aldrich, 99%), Methoxyperfluorobutane (ABCR, 97%), Bromohexane (99%, TCI), Dioctyl sulfosuccinate sodium salt (Sigma Aldrich, 99%), Sodium dodecyl sulfate (Sigma Aldrich, 99%), Zonyl FS-300 (ABCR), Span 20 (ABCR), Zonyl FSE (ABCR), Melamine (Sigma Aldrich, 99%)

Characterization

Zeta potential measurements were performed with a Malvern Panalytical Zetasizer Nano. Atomic force microscopy and related methods were performed in an Oxford Instruments Jupiter XR. Optical power was measured with a Thorlabs PM400 with an calibrated imaging sphere (S142C) at the sample plane.

Deposition of carbon nitride films

Carbon nitride thin films were synthesized according to a previously reported method.[1] Carbon nitride thin films were deposited via chemical vapor deposition (CVD), in a two-heating zones planarGROW-3S-OS CVD using melamine as a precursor. The target substrate (glass slides, 25 mm circles, VWR) are placed in a 3 inch diameter quartz tube directly in the center of the downstream oven. A glass boat containing 5 grams of melamine is placed in the center of the upstream oven. The deposition is done in a vacuum pressure of 10 Torr of nitrogen with 50 sccm of nitrogen flow as carrier gas. Briefly, the temperature at the substrates is increased to 550°C in 40 minutes and, once it reached the final temperature, the upstream oven temperature is raised to 300°C at a rate of 10°Cmin^{-1}. This temperature is held for 30 minutes to quantitatively sublimate the precursor, while the temperature of the downstream oven is kept at 550°C for additional 30 minutes after which the quartz tube is cooled naturally. These samples are removed from the CVD and rinsed before use. The films are not treated additionally, and are hydrophilic upon generation ideal for use with oil-in-water emulsion droplets.

Generation of complex emulsion droplets

Emulsion droplets are composed of a hydrocarbon and fluorocarbon oil with an upper critical solution temperature, which are emulsified in an aqueous phase containing surfactant.[2] Decane (HC oil) and methoxyperfluorobutane (FC oil) are mixed above their upper critical solution temperature (28°C) and emulsified in a heated continuous aqueous phase (containing 3:7 ratio of 1 wt% solutions of sodium dodecyl sulfate and Zonyl FS-300) in an X-junction microfluidic chip (100um) from Dolomite, with fluid pumps purchased from Fluigent. Monodisperse droplets are brought to room temperature for experimentation, after phase-transfer from microfluidic solution to experiment solution via glass pipette. All droplets were used within 48 hours of generation. Droplet morphology characterization is performed utilizing side view optical microscopy, where the geometry of the droplet is used as a descriptor of the balance of interfacial tensions in the surrounding liquid, directly linked to

concentration.[3] The droplet size and internal curvature is corrected for lensing,[4] before calculation of contact angle. Droplets were tested in a variety of sizes, where for comparable behaviors were monodisperse. Tested droplets were in the micrometer-range (10-150 μm).

Full-film excitation of carbon nitride substrate

Carbon nitride-coated glass slides were prepared and utilized with cavity slides to contain complex emulsion droplets, with the carbon nitride facing the liquid sample contained in the well. Prepared samples were placed in a custom microscopic setup, enabling the application of UV light through the glass onto the back of the carbon nitride thin film. The UV light used was 365 nm (Thorlabs), delivered through an optical fiber, and collimated. The experimental setup was retained constant through all experimentation, and the microscope was rotated between sideview and vertical configurations to enable a constant and comparable lighting setup.[5] The behavior was verified to work solely with ultraviolet light, where blue (460 nm), green (530 nm), red (660 nm) and infrared (940 nm) lights of comparable power (~6-8mW, Thorlabs) were utilized and found to be non-interactive with sample or droplets. For experiments, the sample was therefore illuminated with non-interacting green or red light (530nm or 640nm LED) for visualization. For structural color demonstrations, continuous white light was used to illuminate the droplets. The microscope is retained in darkness to avoid ambient light contamination. Carbon nitride thin films were cleaned thoroughly in between uses with water, ethanol, and finally dried with light airflow. The thin films were scratched over time due to normal sample handling, leading to de-lamination of the thin film with the substrate. Each comparable dataset (one figure) was conducted with a singular thin film for comparability, to eliminate possible variation from thin-film thickness.

Anisotropic excitation of carbon nitride substrate

Samples, including droplet-containing samples, were prepared in cavity slides with carbon nitride-thin film glass slides containing them. These prepared samples were placed in a custom microscope, where a 10x objective lens was used to direct ultraviolet light (365nm LED) to the substrate in a designated spot, while sample illumination was done with non-interacting light (530nm or 640nm LED). The custom-built microscope enables rotation of the sample stage, which allows matching experimental conditions when applying UV light to the droplets in a standard top-view, or side-view mode. The objective was mounted on an x-y stage (Thorlabs) which enabled movement of the light spot around the sample, for the manipulation of single and double-phase emulsion droplets. The microscope spot size was retained at uniform for comparable experiments.

Kelvin probe force microscopy

Kelvin probe force microscopy (KPFM) enabled the measurement of charge on the carbon nitride thin film. A carbon nitride thin film coated glass slide was mounted in a custom-built setup to enable application of ultraviolet light from beneath the sample

during AFM measurements. The setup was constructed from a polished block of aluminum with fork to enable the reflection of ultraviolet light (365nm LED) through the glass slide to the carbon nitride thin film, while also grounding the sample for KPFM measurements. All measurements were conducted in an Oxford Instruments Jupiter XR, with gold coated diamond tips (AD-2.8-AS, Oxford Instruments) with a spring constant of 1 to 4.6 N/m with a gold coating. Measurements were conducted in ambient conditions. KPFM was performed with a voltage of 0.5V. Measurements were taken continuously over a period of several hours, and for the purpose of time-resolved measurements, the average of each frame was taken to represent the surface potential per unit time, via custom script in matlab. The data is presented as-recorded and is non-normalized or corrected, and is quantitative as a relative measurement but not as an absolute measurement of surface potential. The atomic force microscope was darkened to prevent light leakage from the environment, and samples were handled in darkness or near darkness prior to measurement, including disabling in-built optical microscope lamp.

Droplet movement and flow characterization

Droplet videos were collected and analyzed with a modified script, in MATLAB, based off of the algorithm of Crocker and Grier modified to track droplets.[6] Videos were analyzed and data taken as movement speed over distance time. Droplet movement was taken for separated, freely-moving droplets and uniform distances from the beam over a uniform time period (10 seconds of movement). For single phase droplet experiments, droplets were composed of a 1:3 mixture of decane and bromohexane, we opted for this specific combination due to the higher density of bromohexane with respect to water, and AOT was used due to the low critical micelle concentration due to SDS. For complex emulsion droplet movement, droplets composed of decane and methoxyperfluorobutane were used, generated in microfluidics, stabilized in solutions of SDS and Zonyl.

Particle image velocimetry was performed utilizing inert tracer microspheres (1 μm Polystyrene, P5olybead) particles in solution which were stable over medium timescales. Videos of particle behavior were characterized using the tool PIVLab from Thielke and Stamhuis.[7] The microscope was focused to the front of a restricted droplet, to visualize surface flows over the body of the droplet in sideview. Velocity flows were calculated from example video with the following settings, FFT multi-pass with integration areas 256, 128, and 64.

Characteristics of thin film adsorption of sodium dodecyl sulfate as measured by droplets:

Time-resolved side view microscopy images of complex emulsion droplets in known concentrations of sodium dodecyl sulfate and Zonyl FS-300 were taken during illumination of the thin films. To measure or understand the adsorption of any surfactant to the substrate, we generate a calibration curve of droplet morphologies in known quantities of surfactant solution (Supporting figure 4). Using this relationship, it is then possible to take a change in droplet morphology and relate it

to a change in the apparent surfactant concentration in solution. This measurement is mediated by the diffusion of molecules, and surfactant exchange with the droplets. In the manuscript, we observe that by charging the carbon nitride thin film, the available ionic surfactant concentration lowers, and by placing the sample in darkness, the apparent ionic surfactant concentration raises. As a note between the relationship of droplet morphology change and the change in surfactant gradient, as concentration gradient of ionic molecules changes over time until it reaches its final position, the deviation of droplet morphologies is also raised. We can use this information to estimate the final amount of adsorbed surfactant, using measurements at different concentrations, which display that a uniform concentration of surfactant is removed from solution. In the manuscript, we measure that the average change of contact angle is $\Delta\theta=16.26°$. Using the calibration curve, this correlates to a change in surfactant concentration of Δc = 0.0121 WT% of a 1 WT% solution of sodium dodecyl sulfate. As the sample holder contained approximately 69µL of solution (sample holder used for each experiment), we can estimate that the mass of SDS which was removed from the solution is 0.78 micrograms of SDS, or 2.7 nanomoles, this translates to 1.63 10^{15} molecules of SDS immobilized from solution by the charged carbon nitride thin film. The carbon nitride thin films are deposited onto 25mm circular glass slides, while in the sample holder only a 20mm diameter circle is exposed to the solution. Assuming that due to the concentrations of SDS to be below the critical micelle concentration, the packing of adsorbed molecules would be in a monolayer, and that they pack to a flat plane on the carbon nitride thin film, we can take the area of the exposed surface (3.1 10^{14} nm^2), and refer these packings to previously measured packing densities of sodium dodecyl sulfate onto surfaces between 2-5 molecules/nm^2, which places the total amount of molecules packed into a monolayer of 1.7 10^{15} molecules of SDS. Compared to our estimated adsorption from the continuous phase, our droplets estimate 103.5% adsorption of molecular SDS to the substrate. We note that major sources of error at these scales can be attributed to human error (pipetting), and the active and illuminated areas of the substrate, which would account for the over-estimation of adsorbed SDS. We can use this information to understand comparable time-dependent adsorption in molecules per square nanometer, where previous reports have demonstrated that sodium dodecyl sulfate will adsorb at approximately one molecule per charge (Supporting figure 9).[8]

Supporting figures

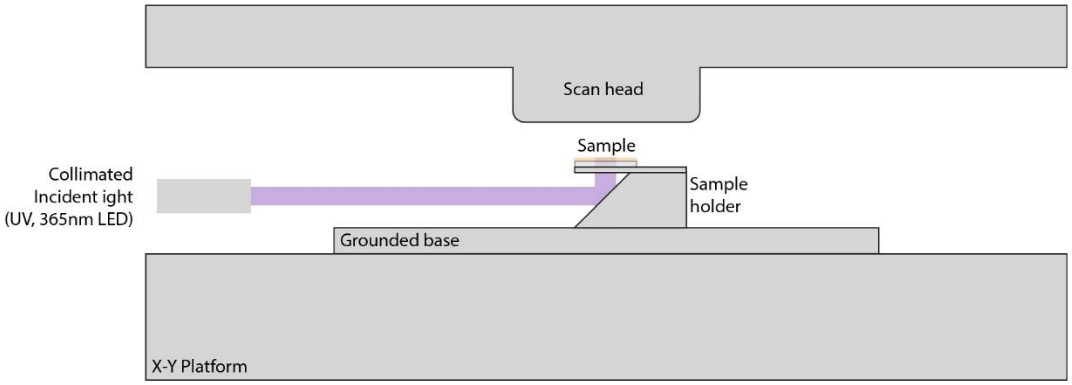

Supporting figure 1. Diagram for the custom sample holder for Kelvin Probe Scanning Microscopy of thin films with light shining through the sample for illumination. The sample holder is machined and polished aluminum composed of the base, and forked sample holding. All parts are attached via silver paste to ensure proper grounding.

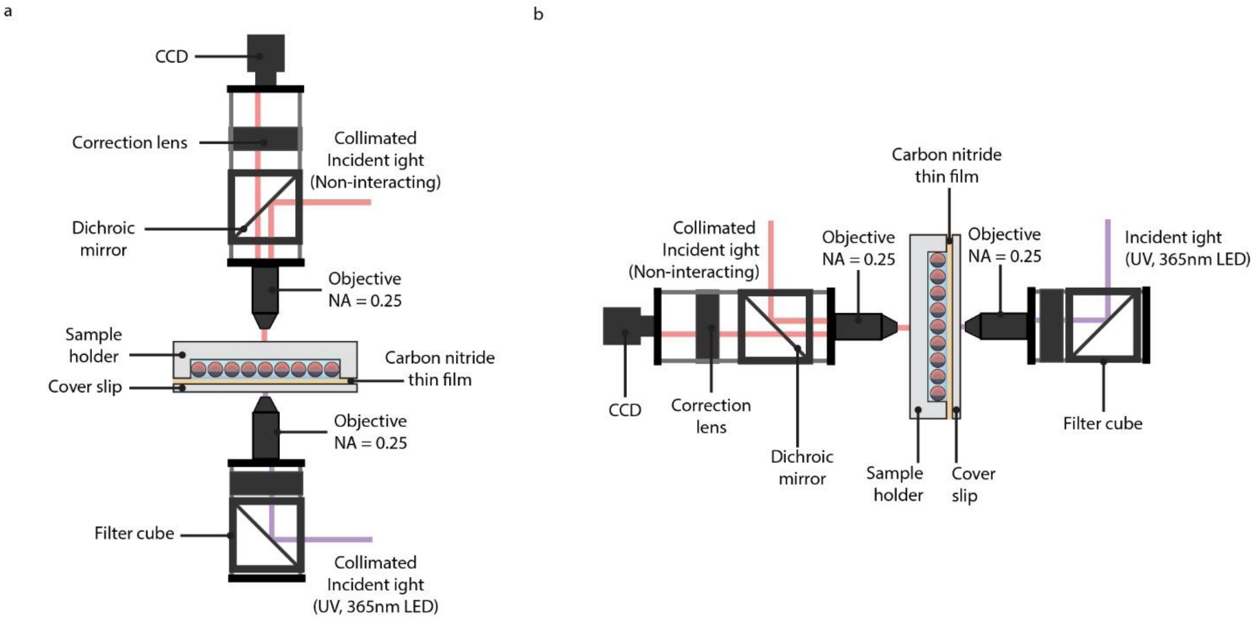

Supporting figure 2. Optical setup for the observation of complex emulsions with anisotropic light delivery, where the custom microscopy setup is mounted on aluminum rails, which are mounted to a rotating stage. Complex emulsion droplets align with gravity, enabling observation of variable behaviors in either the vertical configuration (a) or in the rotated configuration (b). Light is collimated and focused through an objective for delivery in a spot. The light-delivery objective is rotated on an X-Y stage and is retained in a uniform distance for sample-sample comparability.

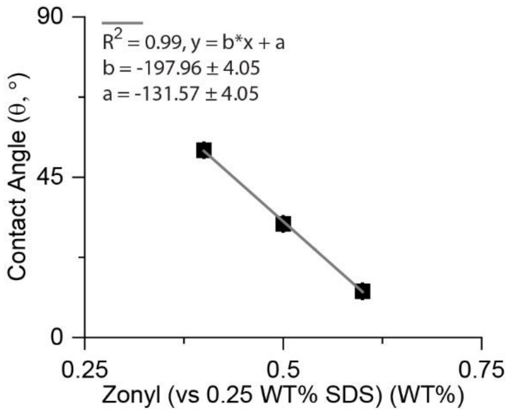

Supporting figure 3. Calibration curve for droplets composed of decane and methoxyperfluorobutane placed in aqueous surfactant solutions with a constant amount of sodium dodecyl sulfate (SDS) at 0.25 WT%, and a variable amount of Zonyl FS-300 (Zonyl). Scalebars represent n=5 measured droplets.

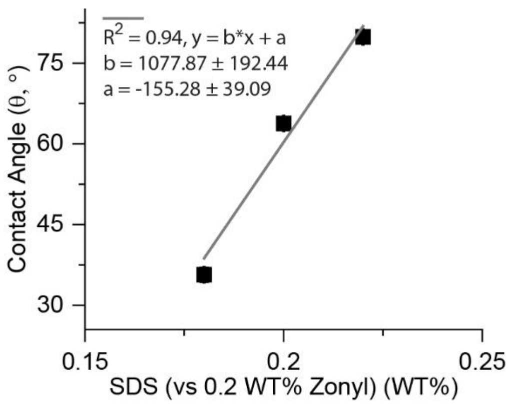

Supporting figure 4. Calibration curve for droplets composed of decane and methoxyperfluorobutane placed in aque ous surfactant solutions with a constant amount of Zonyl FS-300 (Zonyl) at 0.2 WT%, and a variable amount of sodium dodecyl sulfate (SDS). Scalebars represent n=5 measured droplets.

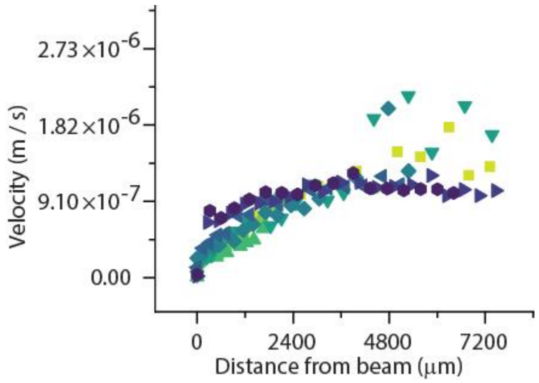

Supporting figure 5. Velocity versus distance from the beam for droplets composed of decane:bromohexane (for density) in a solution of AOT (0.02 WT%), displaying the change in the force gradient acting on separated droplets (color, shape), as droplets get closer to the beam.

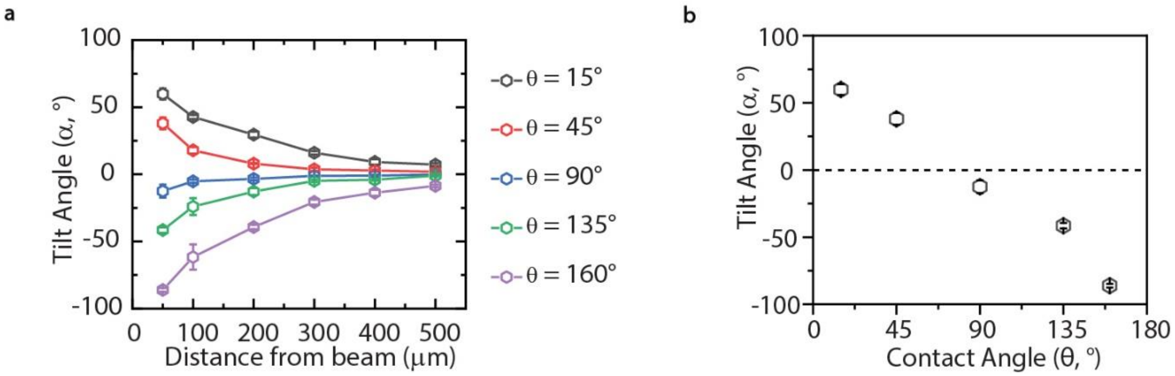

Supporting figure 6. Comparison of droplet tilting for various morphologies at different distances from the beam. Anisotropic fluid flows induced by the interfacial tension differential across the droplet interface further caused a torque (τ) with respect to the droplet center of gravity (CM), resulting in repeatable distance-dependent actuation of the complex droplets. Comparing the maximum tilt angles of different droplet morphologies at minimum beam distance under otherwise uniform conditions, it was found that the droplet tilting is anisotropic and preferential for droplets in hydrocarbon-dominant morphologies. a) Droplet tilt angle versus distance from the beam for complex emulsion droplets in various morphologies. b) Droplet tilt angle 50μm from the beam versus contact angle, displaying an anisotropic tilt profile indicating binary gradients in solution. As the maximum tilt angle for equal-but-opposite morphologies of droplets are drastically different, especially for droplet morphologies where the hydrocarbon-water (ionic surfactant-stabilized) interface are exposed, which interact constructively or destructively with thermal gradients in solution.

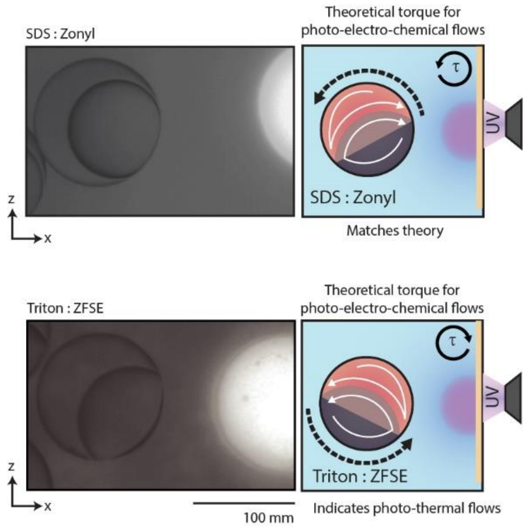

Supporting figure 7. Tilting behavior of droplets indicates thermal, as well as photo-electro-chemical flows around the droplet. For droplets in SDS:Zonyl, where the hydrocarbon surfactant is ionic, droplets tilt as would be expect for ‘purely’ chemical flows. If the ionic surfactant is switched, as with Triton and Zonyl FSE, where the ionic surfactant is now primarily on the fluorocarbon-water interface, we observe that the droplet tilt-angle does not switch, indicating that the dominant factor inducing tilting behavior is heat, and not chemical gradient driven flows.

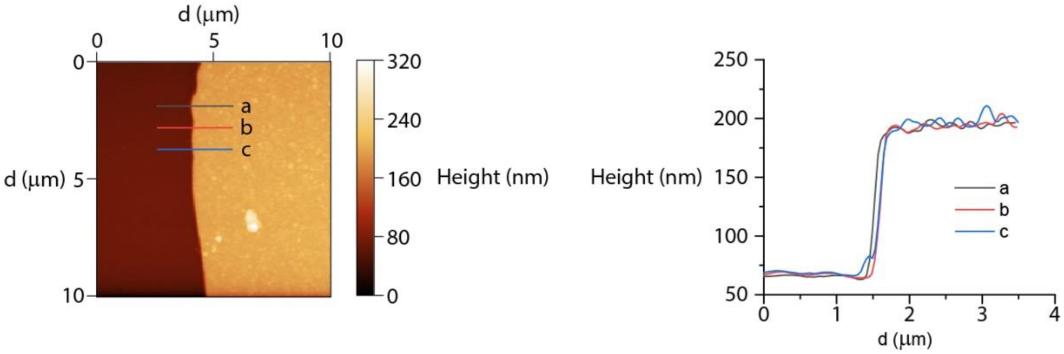

Supporting figure 8. Film characteristics of polymeric carbon nitride synthesized via chemical vapor deposition, including 10 micrometer region step-height map, and three-line traces. The film was partially removed from the substrate with a scratch enabling to visualize, by means of step-height measurements, the thickness of the pCN thin film (120 nm).

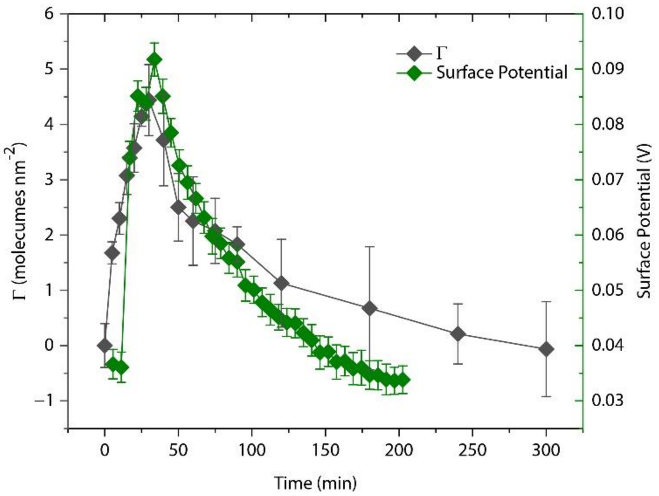

Supporting figure 9. Time-dependent adsorption of SDS to carbon nitride thin film in molecules per square nanometer, compared to the time-dependent surface potential as measured with Kelvin probe force microscopy (KPFM) in ambient conditions (without liquid) limiting comparability. The SDS adsorption in molecules per square nanometer is calculated from calibration curves outlined in the methods section due to the morphological reconfiguration of the droplets, and the sample holder. The surface potential over time is plotted for comparison from the main text (Figure 1). The presence of surface charges affect the dielectric constant, and the interaction between charges and polarizable units, here sodium dodecyl sulfate molecules, cases a delay in the electron-hole recombination, while charging is less affected by the surrounding environment.[8]

References

(1) Giusto, P.; Cruz, D.; Heil, T.; Arazoe, H.; Lova, P.; Aida, T.; Comoretto, D.; Patrini, M.; Antonietti, M. Shine Bright Like a Diamond: New Light on an Old Polymeric Semiconductor. *Adv. Mater.* **2020**, *32* (10), e1908140. DOI: 10.1002/adma.201908140.

(2) Zarzar, L. D.; Sresht, V.; Sletten, E. M.; Kalow, J. A.; Blankschtein, D.; Swager, T. M. Dynamically reconfigurable complex emulsions via tunable interfacial tensions. *Nature* **2015**, *518* (7540), 520-524. DOI: 10.1038/nature14168.

(3) Djalali, S.; Frank, B. D.; Zeininger, L. Responsive drop method: quantitative in situ determination of surfactant effectiveness using reconfigurable Janus emulsions. *Soft Matter* **2020**, *16* (46), 10419-10424, 10.1039/D0SM01724H. DOI: 10.1039/d0sm01724h.

(4) Nagelberg, S.; Zarzar, L. D.; Nicolas, N.; Subramanian, K.; Kalow, J. A.; Sresht, V.; Blankschtein, D.; Barbastathis, G.; Kreysing, M.; Swager, T. M.; et al. Reconfigurable and responsive droplet-based compound micro-lenses. *Nat. Commun.* **2017**, *8*, 14673. DOI: 10.1038/ncomms14673.

(5) Frank, B. D.; Nagelberg, S.; Baryzewska, A. W.; Marqués, P. S.; Antonietti, M.; Kolle, M.; Zeininger, L. Morphology-Directed Light Emission from Fluorescent Janus Colloids for Programmable Chemical-To-Optical Signal Transduction. *Adv. Opt. Mater.* **2023**, 2300875. DOI: 10.1002/adom.202300875.

(6) Crocker, J. C.; Grier, D. G. Methods of digital video microscopy for colloidal studies. *J. Colloid Interface Sci* **1996**, *179* (1), 298-310. DOI: 10.1006/jcis.1996.0217.

(7) Thielicke, W.; Stamhuis, E. J. PIVlab-time-resolved digital particle image velocimetry tool for MATLAB. *J. Open Res. Softw.* **2014**, *7* (0.246), R14. DOI: 10.5334/jors.bl.

(8) Giusto, P.; Kumru, B.; Zhang, J. R.; Rothe, R.; Antonietti, M. Let a Hundred Polymers Bloom: Tunable Wetting of Photografted Polymer-Carbon Nitride Surfaces. *Chem. Mater.* **2020**, *32* (17), 7284-7291. DOI: 10.1021/acs.chemmater.0c01798.

Supporting Movies

Supporting movie 1. A carbon nitride thin film damaged by use is illuminated by ultraviolet (365 nm) light where an air-bubble is either sitting in pure water (left) or in surfactant solution (right). Only the surfactant-containing sample can be seen to dewett, indicating interaction between the charged substrate and the ionic surfactant in solution.

Supporting movie 2. Single-phase droplets composed of decane and bromohexane (1:3) for density and viscosity are placed in a solution of 0.02 wt % AOT, and are observed to move in response to the application of UV light to the carbon nitride thin film which they are placed, the video speed is 600x.

Supporting movie 3. Complex emulsion droplets composed of decane and methoxyperfluorobutane placed on a carbon nitride thin film in an aqueous surfactant solution containing SDS and Zonyl with a morphology that is fluorocarbon dominant (CA=30°) are observed to tilt toward the light spot and move away from the beam. Video is in real time. The light spot is turned off and on to demonstrate the reaction to the application of the light.

Supporting movie 4. Complex emulsion droplets composed of decane and methoxyperfluorobutane placed on a carbon nitride thin film in an aqueous surfactant solution containing SDS and Zonyl as well as tracer particles. The droplets are tuned to have a morphology that is fluorocarbon dominant (CA=30°) and are observed to tilt toward the light spot and move away from the beam. Video is in real time. The light spot is moved to demonstrate the reaction to the light.

Supporting movie 5. Complex emulsion droplets composed of decane and methoxyperfluorobutane are place in a side view optical microscope on a platform and are obstructed from moving. These droplets are in an aqueous surfactant solution containing SDS and Zonyl as well as tracer particles, and are tuned in morphology such that the droplet contact angle is 60°. On application of the UV light spot, flows are visible at the hydrocarbon-water and fluorocarbon-water interface and moving in opposite directions.

Supporting movie 6. Complex emulsion droplets composed of decane and methoxyperfluorobutane are placed in a side view optical microscope at the bottom of a cavity slide in a monolayer. These droplets, in an aqueous surfactant solution composed of SDS and Zonyl are tilting with distance-dependence to the light spot, as demonstrated by moving the light spot (Ultraviolet, 365nm).
